# Reproductive Urology Consultation Is an Opportunity to Evaluate for Medical Comorbidity: The Prevalence of Elevated Blood Pressure and Obesity at Fertility Evaluation

**DOI:** 10.7759/cureus.57071

**Published:** 2024-03-27

**Authors:** Daniel R Greenberg, Evan J Panken, Mary Kate Keeter, Nelson E Bennett, Robert E Brannigan, Joshua A Halpern

**Affiliations:** 1 Department of Urology, Northwestern University Feinberg School of Medicine, Chicago, USA

**Keywords:** infertility, semen analysis, obesity, elevated blood pressure, hypertension

## Abstract

Purpose

To evaluate the prevalence of elevated blood pressure (EBP), hypertension (HTN), and obesity among men presenting for fertility evaluation.

Methods

We retrospectively evaluated all men presenting for male infertility consultation at a single institution from 2000 to 2018. Blood pressure (BP) measurements were abstracted from the electronic health record, and EBP/HTN was defined according to American Heart Association/American College of Cardiology guidelines (systolic blood pressure (SBP) ≥ 120 mmHg or diastolic blood pressure (DBP) ≥ 80 mmHg). Descriptive statistics were used to compare demographic and clinical characteristics of men with and without EBP/HTN or obesity (BMI ≥ 30 kg/m^2^), and logistic regression was utilized to determine associations with EBP/HTN.

Results

Among 4,127 men, 1,370 (33.2%) had a recorded SBP and DBP within one year of their initial visit. EBP/HTN was noted in 857 (62.6%) men. A total of 249 (18.2%) men were obese, 863 (63.0%) were non-obese, and 258 (18.8%) did not have BMI recorded. HTN and obesity were jointly present in 195 (17.5%) men. There was no significant difference in age, ethnicity, or total motile sperm count between men with and without EBP/HTN. On multivariable analysis, BMI was significantly associated with EBP/HTN (OR: 1.13, 95% CI: 1.08-1.18, p < 0.001).

Conclusion

More than half of men presenting for initial fertility consultation have either EBP, obesity, or both. Reproductive urologists should consider routinely screening for these conditions and encourage men to seek further evaluation and treatment, when appropriate.

## Introduction

Male infertility is associated with, and is a harbinger of, medical comorbidity. Men presenting with infertility have a higher risk of diabetes, cardiovascular disease, cancer, and even death [1,2]. As such, reproductive urologists possess a unique opportunity to screen and identify men who are at high risk for developing chronic medical conditions. However, the initial evaluation of the infertile male is quite focused with emphasis on a limited physical exam, serum hormone levels, and semen parameters [3]. Current professional societal guidelines do not recommend screening to identify broader, comorbid conditions in men presenting with infertility.

Vital signs, height, and weight are easily captured data points that can provide invaluable information in the clinical setting, aiding in the triage of men who might require further evaluation. Prior studies have demonstrated that body mass index (BMI) is inversely correlated with semen parameters, rendering this metric useful both for infertility evaluation and comorbidity screening [4]. Cazzaniga et al. demonstrated that a small but significant proportion of European men presenting to a tertiary infertility clinic had elevated blood pressure (EBP) when considering a threshold of 140/90 mmHg [5]. However, in the United States, guidelines from the American Heart Association (AHA)/American College of Cardiology (ACC) define normal blood pressure (BP) as systolic blood pressure (SBP) < 120 mmHg and diastolic blood pressure (DBP) < 80 mmHg, EBP as SBP = 120-129 mmHg and DBP < 80 mmHg, and hypertension (HTN) as SBP ≥ 130 mmHg or DBP ≥ 80 mmHg. HTN is further dichotomized into Stage I, defined as SBP = 130-139 mmHg or DBP = 80-89 mmHg, or Stage II, defined as SBP ≥ 140 mmHg or DBP ≥ 90 mmHg. Utilizing these criteria, we hypothesized that a substantially higher proportion of American men presenting with infertility have EBP or HTN.

Therefore, we sought to identify the proportion of men presenting to a tertiary infertility clinic with EBP/HTN or obesity to determine whether BP and BMI measurement could serve as high-yield screening tools for medical comorbidity in men presenting for initial fertility evaluation.

## Materials and methods

We performed a retrospective analysis of all men presenting for initial male infertility consultation with a single reproductive urologist at a tertiary care center from 2000 to 2018. All patients seen for fertility evaluation, even those without prior semen analysis or documented infertility, were included in the initial analysis. This study was approved by the Institutional Review Board (IRB) at our institution (IRB #00208030).

BP measurements were abstracted from the most proximate visit to fertility consultation within the entire healthcare system. These measurements are not routinely obtained at initial fertility consultations at our institution. This was the motivation for the current retrospective study. Men without BP measurements or those with measurements > one year prior to fertility consultation were excluded from the analysis. Men were stratified according to normal BP or EBP/HTN, which was defined according to AHA/ACC guidelines [6].

Demographics, including age and ethnicity, were obtained for all patients. BMI was recorded for each patient at the initial fertility consultation, and patients were stratified as obese (BMI ≥ 30) or non-obese (BMI < 30). Obesity was further categorized according to the WHO standards as obese class I (BMI ≥ 30 and <35), obese class II (BMI ≥ 35 and <40), and obese class III (BMI ≥ 40) [7]. Semen parameters were obtained, when available, and men were classified as non-oligospermic or oligospermic according to sperm concentration ≤15 million/mL per the WHO 2010 guidelines [8]. Additionally, total motile sperm count (TMSC) was categorized as >20 million, 5-20 million, and <5 million, as these categories have been previously shown to correlate with patients as fertile vs. subfertile vs. requiring in vitro fertilization/intracytoplasmic sperm injection for infertility, respectively [9,10].

Descriptive statistics were utilized to characterize the prevalence of EBP/HTN and obesity. Non-parametric testing was utilized to compare baseline characteristics of men with and without EBP/HTN. Categorical variables were analyzed using chi-square analysis, and continuous variables were analyzed using the Mann-Whitney U test, where appropriate. Univariable and multivariable logistic regression was performed to determine associations between EBP/HTN and patient demographics and semen parameters. Covariates for multivariable analysis were chosen either a priori (as previously identified factors shown to affect semen parameters) or following significance on univariable analyses. These included age, BMI, serum testosterone, ejaculate volume, sperm motility, and TMSC [11]. All statistical analyses were performed using Stata SE version 18 (StataCorp LLC, College Station, TX). All tests of significance were two-sided, and statistical significance was determined at a level of p < 0.05.

## Results

Among 4,127 men presenting for initial fertility consultation, 1,370 (33.2%) had a recorded SBP and DBP within one year of their initial fertility consultation and were included in the analysis. Median SBP and DBP were 120 (interquartile range (IQR): 112-130) and 76 (IQR: 70-80), respectively. EBP/HTN was noted in 857 (62.6%) men, of which 193 (22.5%) were characterized as EBP, 443 (51.7%) as stage I hypertension, and 221 (25.8%) as stage II hypertension.

Among the 1,370 men for whom BP was available, 249 (18.2%) were obese, 863 (63.0%) were non-obese, and 258 (18.8%) did not have BMI recorded. Among obese men, 134 (53.8%) were obese class I, 55 (22.1%) were obese class II, and 60 (24.1%) were obese class III. EBP/HTN and obesity were jointly present in 195 (17.5%) men, whereas EBP/HTN alone was present in 45.5% of men, obesity was present alone in 4.9% of men, and 357 (32.1%) had neither EBP/HTN nor obesity. There was no difference in TMSC or prevalence of oligospermia between obese and non-obese men (p = 0.45 and p = 0.91, respectively).

Characteristics of men with and without EBP/HTN are presented in Table 1. Median BMI was significantly higher for men with EBP/HTN versus men without EBP/HTN (27.3, IQR: 25.0-30.7 vs. 25.5, IQR: 23.4-27.9, p < 0.001). There was no significant difference in age (p = 0.37), ethnicity (p = 0.33), median TMSC (42.0 million, IQR: 6.9-110.6 vs. 49.0 million, IQR: 8.9-107.1, p = 0.81), ejaculate volume, sperm concentration, motility, morphology, or oligo- or azoospermia between men with and without EBP/HTN. Serum testosterone was significantly lower among men with EBP/HTN (312 ng/dL, IQR: 245-405 vs. 339 ng/dL, IQR: 259-424, p = 0.026), and a higher prevalence of men with serum testosterone <300 ng/dl was found among men with EBP/HTN compared to men without EBP/HTN (45.3% vs. 37.0%, p = 0.007).

**Table 1:** Comparison of men with normal BP versus EBP/HTN presenting for fertility evaluation. * Subset n = 1,112; ^ subset n = 744; ~ subset n = 1,063 men. Categorical variables are shown as n (%). Continuous variables are shown as median (interquartile range). P-value < 0.05 was deemed statistically significant. BMI = body mass index; BP = blood pressure; EBP = elevated blood pressure; HTN = hypertension; M = million; T = testosterone.

Characteristic	Total (N = 1370)	Elevated BP/HTN (n = 857)	Normal BP (n = 513)	p-value
Age (years)	35 (31-39)	35 (31-40)	35 (31-39)	0.37
Ethnicity				0.33
Hispanic	74 (5.4%)	46 (5.4%)	28 (5.5%)	
Non-Hispanic	929 (67.8%)	568 (66.3%)	361 (70.4%)	
Declined/unknown	367 (26.8%)	243 (28.4%)	124 (24.2%)	
BMI*	26.5 (24.3-29.6)	27.3 (25.0-30.7)	25.5 (23.4-27.9)	<0.001
Non-obese	863 (77.6%)	506 (72.2%)	357 (86.9%)	
Obese class I	134 (12.1%)	97 (13.8%)	37 (9.0%)	
Obese class II	55 (5.0%)	45 (6.4%)	10 (2.4%)	
Obese class III	60 (5.4%)	53 (7.6%)	7 (1.7%)	
Semen parameters^				
Ejaculate volume (mL)	2.7 (1.8-3.7)	2.7 (1.8-3.7)	2.6 (1.8-4.0)	0.42
Sperm concentration (M/mL)	30.3 (6.6-66.8)	31.5 (7.0-66.0)	30.0 (6.0-68.5)	0.89
Sperm motility (%)	57.0 (40.0-66.0)	58.0 (41.0-66.0)	56.0 (38.5-68.0)	0.82
Sperm morphology (%)	6.0 (2.0-14.0)	7.0 (3.0-14.0)	6.0 (2.0-14.0)	0.51
Oligospermia	265 (35.6%)	169 (36.3%)	96 (34.5%)	0.63
Azoospermia	82 (11.0%)	45 (9.7%)	37 (13.3%)	0.12
Total motile sperm count (M)	45.2 (7.3-109.3)	42.0 (6.9-110.6)	49.0 (8.9-107.1)	0.81
<5 million	161 (21.9%)	101 (21.9%)	60 (21.8%)	
5-20 million	112 (15.2%)	74 (16.0%)	38 (13.8%)	
>20 million	464 (63.0%)	287 (62.1%)	177 (64.4%)	
Total testosterone~ (ng/dL)	321 (250-414)	312 (245-405)	339 (259-424)	0.026
Low testosterone (<300 ng/dL)	448 (42.1%)	297 (45.3%)	151 (37.0%)	0.007

Results from univariable and multivariable logistic regression are presented in Table 2. On univariable analysis, only BMI was significantly associated with EBP/HTN (odds ratio (OR): 1.11, 95% confidence interval (CI): 1.08-1.14, p < 0.001). Likewise, on multivariable analysis, only BMI remained significantly associated with EBP/HTN (OR: 1.13, 95% CI: 1.08-1.18, p < 0.001).

**Table 2:** Univariable and multivariable logistic regression for association with elevated blood pressure or hypertension. P-value < 0.05 was deemed statistically significant. aOR = adjusted odds ratio; BMI = body mass index; CI = confidence interval; OR = odds ratio.

Characteristic	Univariable		Multivariable	
	OR (95% CI)	p-value	aOR (95% CI)	p-value
Age	1.01 (0.99, 1.02)	0.32	0.98 (0.95–1.01)	0.22
BMI	1.11 (1.08, 1.14)	<0.001	1.13 (1.08–1.18)	<0.001
Total testosterone	1.00 (1.00, 1.00)	0.95	1.00 (1.00–1.00)	0.29
Ejaculate volume	0.95 (0.86, 1.04)	0.24	0.94 (0.82, 1.07)	0.35
Sperm motility	1.00 (1.00, 1.01)	0.49	1.01 (1.00, 1.01)	0.28
Total motile sperm count	1.00 (1.00, 1.00)	0.81	1.00 (1.00–1.00)	0.85

## Discussion

Many observational studies have demonstrated a strong relationship between EBP and the risk of both cardiovascular disease (CVD) and chronic kidney disease (CKD), a relationship that until recently was considered strongest among older adults [12]. As such, urologists and men’s health specialists have focused research endeavors and clinical guidelines upon screening for and assessment of cardiovascular risk in men presenting with erectile dysfunction (ED), as these men tend to be older [13-15]. However, two studies in the Journal of the American Medical Association (JAMA) found that EBP in young adult men aged <40 years carries a significantly elevated risk of subsequent CVD [16,17]. As the average age of men presenting with infertility is 35 years, reproductive urologists are uniquely positioned to screen these men for EBP and obesity, serious medical conditions with substantial long-term impact upon morbidity [18].

We found that more than half of all men presenting for infertility evaluation met AHA/ACC criteria for EBP or hypertension. In contrast, Cazzaniga et al. found EBP in 6.8% and 3.6% of infertile men and age-adjusted controls, respectively [5]. The large discrepancy is likely due to the criteria used for EBP/HTN, as the AHA/ACC criteria are more strict. Also, discrepancies in rates of obesity, hypertension, and all-cause mortality between the US and European men likely contribute to our varying results [19,20]. Lastly, BP measurements in the current study were obtained from prior visits within the healthcare system rather than measurements at the time of fertility evaluation. This likely introduced selection bias insofar as these men had prior impetus for interaction with the healthcare system and were, therefore, more likely to have comorbidity. Nonetheless, these studies both suggest a significant prevalence of EBP/HTN in men presenting for infertility evaluation.

EBP/HTN and obesity were jointly present in 17.5% of men, constituting a very high-risk subset of patients. Morbidly obese men aged 25-34 years have a 12-fold increased risk of mortality compared to their non-obese counterparts, and even non-obese overweight men have a higher risk of cardiovascular mortality compared to men with normal weight [21]. Likewise, young men with hypertension have a significantly increased risk of CVD and coronary heart disease-related mortality [16,17,22]. In aggregate, these data reinforce and expand upon a growing body of evidence linking male infertility and comorbidity [2,18,23]. A higher prevalence of oligospermia and azoospermia is seen among men with a higher Charlson Comorbidity Index (CCI), suggesting that subfertility is a proxy for overall health status [24,25]. Although we found no significant difference in the prevalence of oligospermia or azoospermia between cohorts, multiple claims-based studies have found a higher incidence of chronic medical conditions and malignancy among infertile men [2,23].

Beyond the association of infertility and future incidence of comorbidity, some studies have suggested a direct impact of hypertension on semen parameters. Guo et al. found that hypertensive men had significantly impaired semen parameters compared to non-hypertensive controls, and multiple potential mechanisms have been proposed [9,26]. In contrast, Cazzaniga et al. did not find a significant difference in semen parameters between men with and without EBP [5]. Likewise, the present study did not detect a difference in TMSC between these two groups. Further studies are needed to determine whether hypertension can directly impair semen parameters and the mechanisms by which this may occur.

In light of the high prevalence of both hypertension and obesity in men presenting for fertility evaluation, we propose that reproductive urologists consider recording BP and BMI for all men presenting for initial fertility evaluation. With almost 20% of all men in the United States reporting that they do not have a primary care physician, the opportunity to identify and refer a substantial proportion of men at high risk for comorbidity cannot be overlooked [27]. Reproductive urologists are uniquely positioned as medical gatekeepers for young adults who may not otherwise seek medical care, and thus fertility evaluation can serve as an alternative opportunity for compliance with the United States Preventive Services Task Force (USPSTF) blood pressure and obesity screening guidelines [28,29]. Reproductive urologists are therefore positioned to encourage young men with these comorbidities to establish care with a primary care physician to provide longitudinal care and follow-up screening.

The current study must be interpreted within the context of limitations in study design. First, BP was recorded on a single occasion in the clinical setting and was not confirmed on a subsequent encounter. Office BP measurement is less accurate and reproducible than ambulatory or home measurements, and the lack of repeat measurement may have either over- or under-estimated the true prevalence of hypertension, which requires two measurements over time [6]. However, prior data show that repeated BP measurements have minimal intra-patient variability, and the variability that does occur is greatest with SBP > 160 mmHg [30]. Likewise, we did not examine the utilization of anti-hypertensive agents in the cohort. As such, a number of men may have had normal BP measurements resulting from successful treatment of hypertension, which would lead to further underestimation of the true prevalence of hypertension. Second, as mentioned above, measurements were not obtained at the infertility point-of-care; rather, measurements were abstracted from prior visits within the healthcare system, which biases the cohort toward men with pre-existing comorbidity for which they already established medical care. Third, the current study was not limited to men with documented infertility. Instead, we included all men presenting for fertility evaluation, including those ultimately found to have unimpaired fertility potential. Given the known relationship between infertility and comorbidity, it is possible that the proportion of infertile men with EBP is even higher than reported for the overall cohort. We were also unable to compare our cohort to the general population due to the retrospective nature of the study. Lastly, the comparison of semen parameters and BMI is susceptible to bias secondary to missing data in a substantial proportion of patients.

## Conclusions

More than half of the men in our study who presented for initial fertility consultation had either high BP, obesity, or both. These conditions may not only impact fertility but have broader implications for general health status. Given the relatively young age of presentation for fertility evaluation, reproductive urologists should consider routinely screening for these conditions and encourage men to seek further evaluation and treatment, when appropriate, to prevent the sequelae of these underlying risk factors.
